# Obituary

**Published:** 1887-01

**Authors:** 


### EDITORIAL.

### Obituary.

Died at Rising Sun, Indiana, November 9th, 1886, Dr. Joel P. Ulrey, D.D.S. He was born in Lebanon, Warren County, Ohio, May, 1817. His father, Daniel Ulrey, was a Pennsylvanian, and moved with his parents to the Northwestern Territory about the year 1800, having for a time stopped in Kentucky. They purchased a farm in what is now the heart of Cincinnati. Subsequent to this his father turned his attention to boating on the Ohio river. About forty years ago they removed to Rising Sun, Indiana. Dr. Ulrey's mother died in 1869, his father in 1879. The doctor spent his earlier years in the vicinity of Cincinnati. He was educated in the schools of Lebanon, Ohio, where, after closing his school work, he was engaged for several years in a printing office, and subsequently, at the same business in Cincinnati. At the latter place he began the study of dentistry about the year 1836.

Dentistry at this time was in one sense in its infancy; it had no colleges, no journals, and no Associations.

Notwithstanding this condition of things, Dr. Ulrey made remarkable attainments, and wrought out for himself an enviable reputation. He was always found abreast with the foremost in sustaining and pressing forward whatever would promote the interest of his chosen profession.

He was one of the pioneers in the establishment of the Ohio College of Dental Surgery, and throughout his life gave it his hearty support. He received the degree of D.D.S. from the college not many years after its establishment.

He was also one of the organizers of the Mississippi Valley Dental Society, the first organization of the kind in the West, and now the oldest in the world.

The doctor was present at the meeting of this body for about forty successive years. He gave material aid and support to the periodical literature of our profession.

Thus it will be seen, that, though he was quiet in manner, and unostentatious almost to a fault, he did much for the development and progress of dental science and art. He was engaged in the practice of his profession for about fifty years, forty of which he was located at Rising Sun. A peculiar feature of his practice was, that during almost the whole of this forty years he divided each week of his labor equally between three places, namely: Rising Sun, Aurora, and Lawrenceburgh, and it is very possible, that the labor he has taken upon himself, in traveling between these points, during recent years at least, required strength, which, had it been husbanded, would most likely have added to his lease of life.

Dr. Ulrey was a most estimable Christian gentleman, a man of the purest integrity in all of his relations with his fellow man. Highly esteemed by every one who knew him, and those who knew him best, esteemed him most. His name is worthy to be enrolled with those who have stood in the foremost rank of our professional heroes.

